# Impact Evaluation of a System-Wide Chronic Disease Management Program on Health Service Utilisation: A Propensity-Matched Cohort Study

**DOI:** 10.1371/journal.pmed.1002035

**Published:** 2016-06-07

**Authors:** Laurent Billot, Kate Corcoran, Alina McDonald, Gawaine Powell-Davies, Anne-Marie Feyer

**Affiliations:** 1 The George Institute for Global Health, Sydney, New South Wales, Australia; 2 Sydney Medical School, The University of Sydney, Sydney, New South Wales, Australia; 3 Centre for Primary Health Care and Equity, University of New South Wales, Sydney New South Wales, Australia; Stanford University, UNITED STATES

## Abstract

**Background:**

The New South Wales Health (NSW Health) Chronic Disease Management Program (CDMP) delivers interventions to adults at risk of hospitalisation for five target chronic conditions that respond well to ambulatory care: diabetes, hypertension, chronic obstructive pulmonary disease, congestive heart failure, and coronary artery disease. The intervention consists of two main components: (1) care coordination across sectors (acute, ambulatory, and community care from both public and private sectors) and clinical specialties, facilitated by program care coordinators, and (2) health coaching including management of lifestyle risk factors and medications and self-management. These components were broadly prescribed by the head office of NSW Health, which funded the program, and were implemented by regional health services (local health districts) in ways that best suited their own history, environment, workforce, and patient need. We used a propensity-matched cohort study to evaluate health service utilisation after enrolment in the CDMP.

**Methods and Findings:**

The evaluation cohort included 41,303 CDMP participants enrolled between 1 January 2011 and 31 December 2013 who experienced at least one hospital admission or emergency department (ED) presentation for a target condition in the 12 mo preceding enrolment. Potential controls were selected from patients not enrolled in the CDMP but experiencing at least one hospital admission or ED presentation over the same period. Each CDMP patient in the evaluation cohort was matched to one control using 1:1 propensity score matching. The primary outcome was avoidable hospitalisations. Secondary outcomes included avoidable readmissions, avoidable bed days, unplanned hospitalisations, unplanned readmissions, unplanned bed days, ED presentations, and all-cause death.

The primary analysis consisted of 30,057 CDMP participants and 30,057 matched controls with a median follow-up of 15 mo. Of those, 25,638 (85.3%) and 25,597 (85.2%) were alive by the end of follow-up in the CDMP and control groups, respectively. Baseline characteristics (including history of health service utilisation) were well balanced between the matched groups. In both groups, utilisation peaked just before the time of enrolment/matching, declined sharply immediately following enrolment, and then continued to decrease more gradually; however, after enrolment, avoidable and unplanned health service utilisation remained higher for CDMP participants compared to controls. The adjusted yearly rate of avoidable hospital admissions was 0.57 (95% CI 0.52 to 0.62) in the CDMP group versus 0.33 (95% CI 0.31 to 0.37) in the control group (adjusted rate ratio 1.70, 95% CI 1.62 to 1.79, *p <* 0.001). Significant increases in service utilisation were also observed for unplanned hospitalisations (1.42, 95% CI 1.37 to 1.47, *p <* 0.001) and ED presentations (1.37, 95% CI 1.32 to 1.42, *p <* 0.001) as well as avoidable (2.00, 95% CI 1.80 to 2.22, *p <* 0.001) and unplanned (1.51, 95% CI 1.40 to 1.62, *p <* 0.001) readmissions and avoidable (1.70, 95% CI 1.59 to 1.82, *p <* 0.001) and unplanned (1.43, 95% CI 1.36 to 1.49, *p <* 0.001) bed days. No evidence of a difference was seen for all-cause death (adjusted risk ratio 0.96, 95% CI 0.96 to 1.01, *p* = 0.10) or non-avoidable hospitalisations (all hospitalisations minus avoidable hospitalisations; adjusted rate ratio 1.03, 95% CI 0.97 to 1.10, *p* = 0.26).

Despite the robustness of these results to sensitivity analyses, in the absence of a randomised control group, one cannot exclude the possibility of residual or unmeasured confounding that was not controlled for by the matching process and multivariable analyses.

**Conclusions:**

Participation in the CDMP was associated with an increase in avoidable hospital admissions compared to matched controls but no difference in the rate of other types of hospitalisation or death. A possible explanation is that the program identified conditions that required participants to be hospitalised. Service utilisation decreased sharply following its peak for both groups. This finding reflects the natural tendency for high-risk patients to show reductions in use following intense phases of service utilisation and highlights that, despite the additional complexity, a carefully selected control group is essential when assessing the effectiveness of interventions on hospital use.

## Abstract 2

In this propensity-matched cohort study, Laurent Billot and colleagues evaluate health service utilization among patients enrolled in the New South Wales Health Chronic Disease Management Program.

## Author Summary

**Why Was This Study Done?:**

- There is an increasing number of people living with chronic disease and whose chronic conditions lead to increased hospitalisations and expenditures for health systems.
- There is evidence to support the effectiveness of a variety of interventions, including self-management support and care coordination.
- New South Wales Health implemented the Chronic Disease Management Program to help people with chronic disease better manage their conditions in the community and commissioned an independent evaluation to assess the effect of the program on future hospitalisations, especially so-called avoidable, or “potentially preventable”, hospitalisations.

**What Did the Researchers Do and Find?:**

- We compared hospitalisation patterns in 30,057 individuals enrolled in the Chronic Disease Management Program to those in 30,057 similar individuals who were not part of the program and who were matched using a propensity score.
- We found, over an average follow-up of 15 mo, that avoidable hospitalisations appeared more frequent in those enrolled in the program (adjusted rate ratio 1.70, 95% CI 1.62 to 1.79, *p <* 0.001), with most of the excess related to the conditions targeted by the program such as chronic respiratory and cardiovascular diseases.
- Excesses were also observed for other secondary outcomes such as unplanned hospital admissions, readmissions, length of hospital stay, and emergency department presentations, but no difference was seen in mortality or non-avoidable hospitalisations.

**What Do These Findings Mean?:**

- The Chronic Disease Management Program may have uncovered unaddressed needs that led to increased hospitalisations related to the conditions targeted by the program.
- The results were consistent across outcomes and robust in sensitivity analyses; however, due to the non-randomised nature of the evaluation, one cannot exclude the possibility of unmeasured confounding that could not be accounted for by propensity matching and multivariable analyses.
- Other health indicators, such as data collected by general practices, might provide additional insight for the early identification of individuals who might benefit from similar programs, and might provide more sensitive measures of program effectiveness.

## Introduction

Health systems were originally designed largely for the provision of acute and reactive care. They now need to be reoriented towards the care and management of the increasing numbers of individuals living with chronic and complex conditions [1–3]. While there is increasing evidence for the effectiveness of a variety of interventions, including self-management support [4,5] and care coordination [6–8], there is less evidence available on the effectiveness or cost-effectiveness of system-wide implementations [9]. Complex, large-scale interventions are resource-intensive and go against the tendency towards incremental change common to large systems. With the growing momentum for governments to measure the outcomes of programs and increase the transparency of expenditure [10], the ability to measure the impact of a healthcare intervention has become crucial in driving and informing system-wide change [11–13].

A variety of theories of large-scale change in complex health systems have been formulated in the last decade [12]. The UK Medical Research Council formulated key considerations in the evaluation of complex interventions based on traditional experimental methodologies such as randomised controlled trials (RCTs) [14]. More recent thinking recognises that non-experimental methods are also needed and that RCTs may not always be appropriate for evaluating such interventions [9,15,16].

In 2010, a large-scale, complex program was rolled out in the public health services of New South Wales, Australia. New South Wales Health (NSW Health) provides public acute care for a population of approximately 7 million people over an area of 800,642 km^2^ (309,130 square miles), with an annual expenditure of around $20 billion (Australian dollars). Like most health services, NSW Health is facing a growing demand for acute services due to the increasing burden of chronic conditions in the population. NSW Health’s Chronic Disease Management Program (CDMP) aimed to reduce avoidable hospitalisation by improving the management of individuals with chronic conditions. The program involved identifying adults who were at risk of avoidable hospitalisation for one of five target conditions: diabetes, hypertension, chronic obstructive pulmonary disease, congestive heart failure, and coronary artery disease [17]. Program participants were provided self-management support and care coordination to better manage their conditions in the community. The specific arrangements for these services varied across local health districts, within the model prescribed by the head office of NSW Health (See S1 Table).

NSW Health commissioned an independent evaluation aimed at assessing the impact of the CDMP on hospital utilisation, with a focus on avoidable (or “potentially preventable”) hospital admissions. Due to the complex and system-wide deployment of the program, a randomised trial was not possible, and the counterfactual was obtained via propensity matching using state-wide hospital and emergency department (ED) records. This paper reports the findings of the quantitative evaluation.

## Methods

### Ethics Approval

Ethical approval for this project was received from the New South Wales (NSW) Population and Health Services Research Ethics Committee (HREC/11/CIPHS/69). Following the establishment of the CDMP Outcomes Register in mid-2013, individual consent was no longer required for the purpose of data linkage and evaluation. This applied both prospectively and retrospectively, thus allowing the linkage of all individuals enrolled in the CDMP to NSW hospital and ED records.

### Study Design

The primary aim of the CDMP was to help people with chronic diseases better manage their conditions and reduce their need for future hospitalisations. The CDMP intervention consisted of two main components: (1) care coordination across sectors (acute, ambulatory, and community care from both public and private sectors) and clinical specialties, facilitated by program care coordinators, and (2) health coaching including management of lifestyle risk factors and medications and self-management.

The CDMP evaluation was an observational study using linked hospital and ED records and propensity matching to create a comparable control group.

### Study Population

The CDMP targeted individuals with diabetes, congestive heart failure, coronary artery disease, chronic obstructive pulmonary disease, or hypertension [17], aged 16 y or older, who were at high risk of hospitalisation for their chronic condition.

Study participants included all individuals enrolled in the CDMP between 1 January 2011 and 31 December 2013. Potential controls were identified as individuals not enrolled in the CDMP by 31 December 2013 who were hospitalised in NSW between 1 January 2007 and 31 December 2013 for any of the conditions targeted by the CDMP and who had least one hospital admission or ED presentation between January 2010 and December 2013. A matched subset of CDMP participants and potential controls was selected using propensity scoring.

### Data

A CDMP Outcomes Register was established in mid-2013 under the “public health and disease registers” provisions of the Public Health Act 2010 (NSW), providing a mechanism for linking and de-identifying records for program evaluation without requiring individual consent. The register contained linked individual records from the following sources: (1) CDMP minimum dataset: record of all the individuals registered in the CDMP by the local health districts between 1 January 2007 and 31 December 2013; (2) potential controls: record of all individuals who were admitted to hospital with any of the target conditions between 1 January 2007 and 31 December 2013 but were not enrolled in the CDMP; (3) records of the NSW Admitted Patient Data Collection, including all hospital separations (including discharges, transfers, and deaths) from all NSW public and private hospitals and day procedure centres, held by NSW Health, for the period 1 January 2007 onwards that link to either (1) or (2); (4) records of the NSW Emergency Department Data Collection, including all emergency presentations to public hospitals, held by the Ministry of Health, for the period 1 January 2007 onwards that link to (1) or (2); and (5) records of all deaths occurring in NSW, held by the NSW Registry of Births Deaths & Marriages and the Ministry of Health, for the period 1 January 2007 onwards that link to (1) or (2).

A decision was made to exclude individuals registered in the CDMP before 2011. Although the CDMP commenced formally in mid-2010, early stages were taken up with establishing the program (including creating the CDMP minimum dataset) rather than delivering services. By the beginning of 2011, the program was operational across the state.

### Statistical Analysis

The primary outcome was the rate of avoidable (or “potentially preventable”) hospital admissions as defined by the Victorian Department of Human Services and NSW Health based on ICD codes related to a range of vaccine-preventable, chronic, and acute conditions (see S2 Table for details). Secondary outcomes included unplanned hospital admissions, defined as emergency hospital admissions excluding secondary diagnoses of palliative care (ICD code Z51.5) and discharge to palliative care; ED presentations; avoidable readmissions, defined as an unplanned hospital admission followed by an avoidable admission within 28 d of discharge; unplanned readmissions, defined as an unplanned hospital admission followed by another unplanned admission within 28 d of discharge; avoidable bed days; unplanned bed days; National Admitted Weighted Units, a measure of health service activity expressed as a common unit that provides a way of comparing and valuing each public hospital service by weighting it for its clinical complexity; and all-cause mortality. Post hoc outcomes included all hospitalisations and non-avoidable hospitalisations, defined as all hospitalisations minus avoidable hospitalisations. Except for mortality, each outcome was analysed as a yearly rate, calculated as the number of events (e.g., hospitalisations or days) occurring during follow-up. Follow-up time, or exposure, was defined as the number of years between the month of enrolment and 31 December 2013.

Baseline characteristics for the CDMP evaluation cohort and potential controls were obtained using the data collected during hospitalisations and ED presentations during the 12 mo preceding enrolment/matching, including sociodemographic characteristics, recent patterns of service utilisation, and key diagnoses including those targeted by the CDMP. See S1 Text for a full list of baseline characteristics and covariates.

CDMP participants were matched to a subset of potential controls using a propensity score corresponding to the probability of being enrolled in the CDMP [18]. To derive the propensity score, 54 baseline variables were included in a multiple regression model where the outcome was a binary variable indicative of CDMP enrolment. All main effects (baseline variables) were forced into the model while all two-way interactions were selected using automatic forward selection (threshold *p*-value = 0.05). Variables with missing values were included by creating a “missing” category. To qualify as a possible match for a given enrolment month, a participant from the set of potential controls had to have at least one hospitalisation or ED presentation in the 12 mo preceding that month. Depending on their pattern of hospitalisations, potential controls could qualify as a possible match for more than one enrolment month. To remove the need to account for correlations due to repeated measurements as well as to allow the propensity to vary over time, a separate model was derived for every month of enrolment. Due to the automatic variable selection process, all 35 monthly models ended up including a slightly different set of interactions in the propensity calculation.

Each CDMP participant was then matched to one control using one-to-one greedy matching with a caliper of 0.02 using the SAS %gmatch macro [19]. The matching process started with CDMP participants enrolled in month 1 (January 2011), then continued with those enrolled in month 2, month 3, etc., until month 35 (November 2013). Because some controls were potential matches for more than one enrolment month, a control matched to a CDMP participant in an early month was excluded from the matching pool for future months and so was unavailable for matching to another CDMP participant at another time. In the end, each CDMP participant was matched to only one control, who had the same propensity score at the time of CDMP enrolment, and each control was matched to at most one CDMP participant. The quality of the matching was assessed by comparing baseline characteristics before and after matching using standardised differences [20].

All service utilisation outcomes were analysed using Poisson regression with exposure as an offset. We used generalised estimating equations to account for the correlation within matched pairs. Covariates included the effect of the program (CDMP versus control), the month of enrolment/matching (January 2011 to November 2013), and a month-by-program interaction, thus allowing the intervention effect to be estimated separately for each month. The overall intervention effect was obtained by using exponentiated least-squares means that averaged the intervention effect across all months. In addition, covariates included all 54 main effects used in the propensity score calculation as well as the propensity score itself. Although all those variables were already included in the propensity score model, this approach adjusted for residual covariate imbalances between the matched groups following the principle of “double robustness” [20]. To avoid underestimating utilisation rates, only survivors were included in the models for service utilisation outcomes. Mortality was analysed separately as the proportion who died by the end of follow-up.

Sensitivity analyses included the use of a 2-y baseline instead of a 1-y baseline, that is, considering patients who had at least one hospital admission or ED presentation in the 24 mo (instead of 12 mo) preceding their enrolment or matching. Other preplanned analyses (see S1 File for the statistical analysis plan) included “before-after” and “time-dependent” analyses performed in the CDMP evaluation cohort only; however, due to the lack of a counterfactual, these analyses were abandoned in favour of the propensity-matched analyses. There are other minor differences between the final analysis plan and the analysis reported here: (1) readmissions and bed days were split into unplanned and avoidable events, (2) the National Admitted Weighted Units analysis was removed from the final version of the analysis plan due to excessive missing data, and (3) the readmission cutoff was changed from 30 to 28 d following communication from NSW Health that the metric used by local health districts was based on a 28-d cut point. All these changes were made before performing analyses by group.

We performed the following post hoc analyses to confirm the findings of our planned analyses and assist with the interpretation: (1) unmatched multivariable analyses comparing the CDMP evaluation cohort to the entire set of potential controls after randomly selecting 1 mo from each control in order to reduce the size of the dataset as well as remove correlations and (2) longitudinal plots describing the average rate of utilisation per month defined as time from enrolment or matching. Following peer review, an extra set of sensitivity analyses was performed: (1) an analysis of time to death with a Cox model adjusting for the same variables as the Poisson regressions and using the robust sandwich covariance estimate [21] to account for the correlation within matched pairs, (2) a breakdown of hospitalisations pre- and post-enrolment/matching per ICD category, and (3) a new set of matched analyses based on a wider set of 54 covariates included both in the propensity score calculation and in the matched Poisson models.

Given the very large sample size, *p*-values smaller than 0.001 were considered significant. All analyses were performed using SAS version 9.2 or above.

## Results

### Data Flow and Baseline Characteristics

**Fig 1:**
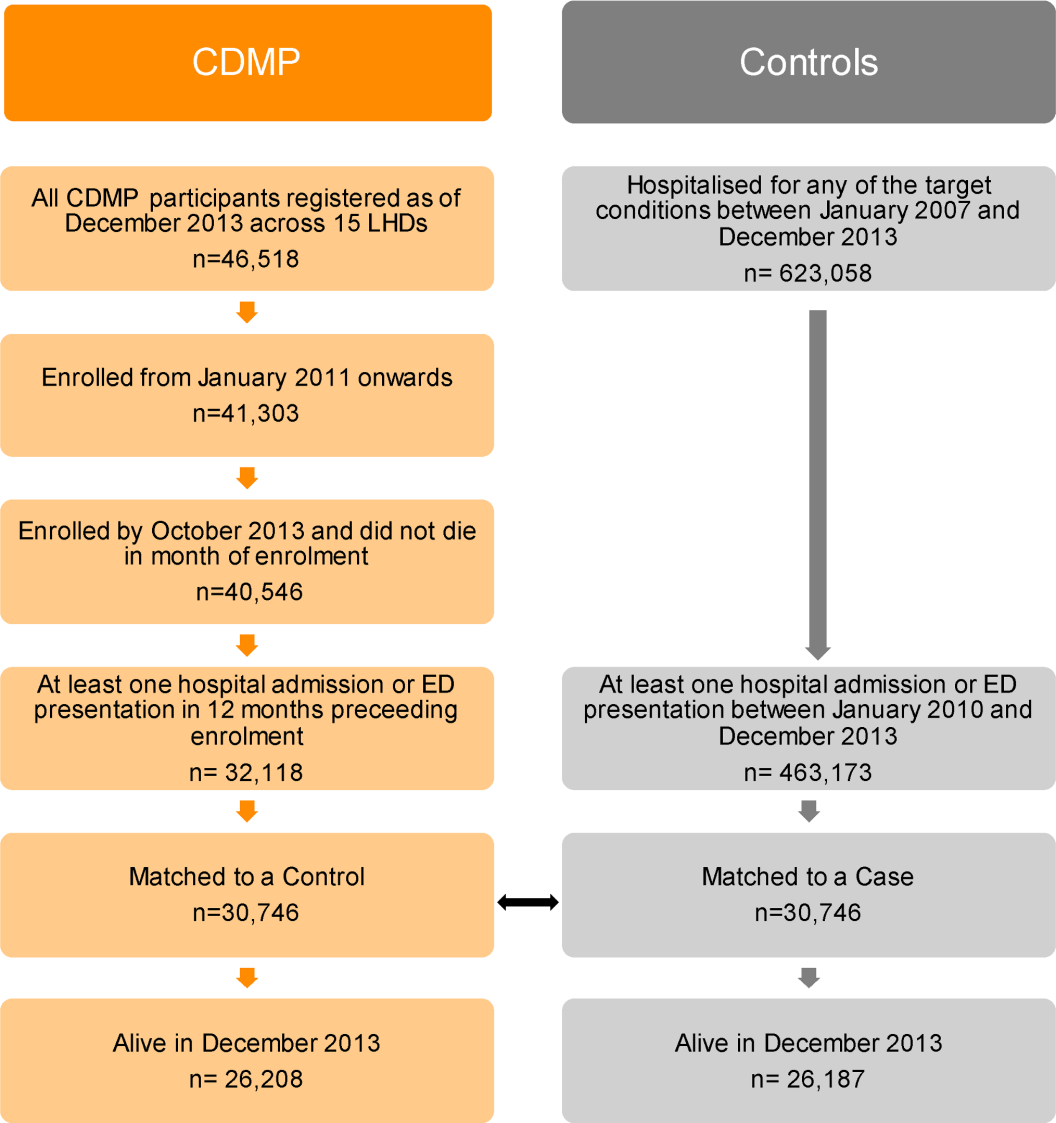
Study flowchart.

A total of 46,518 individuals were registered in the CDMP dataset across 15 local health districts as of 31 December 2013. The cohort used for the evaluation consisted of the 41,303 (88.8%) individuals who had a non-missing enrolment date and were registered from 1 January 2011 onwards (Fig 1). The analysis was further restricted to the 40,546 participants who had the opportunity to be followed up for at least 1 mo, that is, participants enrolled by the end of October 2013 and who were still alive at the end of the month during which they were enrolled. Follow-up time ranged between 0 and 34 mo with a median of 15 mo, where a follow-up time of zero corresponds to patients who died at the beginning of the month following their month of enrolment and a follow-up time of 34 mo corresponds to patients who were enrolled in January 2011 and were still alive on 30 November 2013.

A broad comparison cohort included 623,058 individuals who were hospitalised for one of the target conditions at least once between 2007 and 2013. Of those, 463,173 had at least one hospital admission or ED presentation between 2010 and 2013 and were eligible as potential controls.

The evaluation cohort was evenly split by sex (47.8% males) and included a high proportion of older participants (44.2% aged 75 y or older) (Table 1). Potential controls had a similar age and sex distribution (standardised differences [SDiffs] ≤ 0.10). Around 6% of CDMP participants were Aboriginal or Torres Strait Islanders versus 2.2% among the potential controls; this difference resulted from the inflated enrolment targets set for this population for the CDMP. Before matching, the CDMP evaluation cohort and the potential controls had very different backgrounds of service utilisation. Participants in the CDMP evaluation cohort reported an average of 0.79 (standard deviation [SD] 1.57) avoidable admissions in the 12 mo preceding their enrolment versus a yearly average of 0.26 (SD 0.76) in the potential controls (SDiff = 0.49) in a randomly selected 12-mo period. In addition, except for cerebrovascular diseases, dementia, and obesity, participants in the CDMP evaluation cohort who were hospitalised at least once during the 12 mo preceding their enrolment had a much higher prevalence of co-morbidities than the potential controls hospitalised at least once during the randomly selected 12-mo period. As expected, this was especially true for the conditions targeted by the program, namely, hypertension (2.2% versus 0.8%, SDiff = 0.11), diabetes (19.8% versus 8.5%, SDiff = 0.33), chronic obstructive pulmonary disease (16.3% versus 3.3%, SDiff = 0.45), congestive heart failure (10.9% versus 2.4%, SDiff = 0.35), and coronary artery disease (26.4% versus 13.3%, SDiff = 0.33).

**Table 1:** Baseline characteristics before and after matching.

Characteristic	Before Matching			After Matching		
	CDMP Evaluation Cohort (*n* = 41,303)	Potential Controls (*n* = 463,173)	SDiff	Matched CDMP Group (*n* = 30,057)	Matched Control Group (*n* = 30,057)	SDiff
**Age** ^1^	*n* = 39,184	*n* = 463,171		*n* = 30,057	*n* = 30,057	
<50 y	3,710 (9.5%)	43,450 (9.4%)	0.00	2,646 (8.8%)	2,805 (9.3%)	0.02
50 to <65 y	8,160 (20.8%)	115,886 (25.0%)	0.10	6,042 (20.1%)	6,706 (22.3%)	0.05
65 to <75 y	9,981 (25.5%)	119,027 (25.7%)	0.01	7,708 (25.6%)	7,289 (24.3%)	0.03
≥75 y	17,333 (44.2%)	184,808 (39.9%)	0.09	13,661 (45.5%)	13,257 (44.1%)	0.03
**Sex** ^1^	*n* = 41,241	*n* = 463,156		*n* = 30,057	*n* = 30,057	
Male	19,694 (47.8%)	216,171 (46.7%)	0.02	14,189 (47.2%)	14,030 (46.7%)	0.01
Female	21,547 (52.2%)	246,985 (53.3%)		15,868 (52.8%)	16,027 (53.3%)	
**Aboriginal or Torres Strait Islander** ^1^	*n* = 41,283	*n* = 463,173		*n* = 30,057	*n* = 30,057	
Yes	2,423 (5.9%)	9,983 (2.2%)	0.19	1,831 (6.1%)	1,448 (4.8%)	0.06
No	38,860 (94.1%)	453,190 (97.8%)		28,226 (93.9%)	28,609 (95.2%)	
**Marital status** ^1^	*n* = 0	*n* = 463,173		*n* = 30,057	*n* = 30,057	
Married	n/a	231,686 (50.0%)	n/a	13,923 (46.3%)	14,354 (47.8%)	0.03
Never married	n/a	51,662 (11.2%)	n/a	3,593 (12.0%)	3,567 (11.9%)	0.00
Widowed	n/a	84,813 (18.3%)	n/a	6,411 (21.3%)	6,330 (21.1%)	0.01
Divorced/separated	n/a	85,847 (18.5%)	n/a	5,765 (19.2%)	5,366 (17.9%)	0.03
Unknown/missing	n/a	9,165 (2.0%)	n/a	365 (1.2%)	440 (1.5%)	0.02
**Service utilisation (last 12 mo)** ^2^	*n* = 41,303	*n* = 463,173		*n* = 30,746	*n* = 30,746	
Any avoidable admission	16,955 (41.1%)	88,417 (19.1%)	0.49	15,382 (51.2%)	15,444 (51.4%)	0.00
Number of avoidable admissions^3^	0.79 (1.57)	0.26 (0.76)	0.43	0.95 (1.39)	1.00 (1.95)	0.03
Any unplanned admission	26,355 (63.8%)	220,277 (47.6%)	0.33	24,478 (81.4%)	22,807 (75.9%)	0.13
Number of unplanned admissions^3^	1.49 (1.91)	0.68 (1.23)	0.50	1.85 (1.78)	1.74 (2.33)	0.06
Any hospital admission	30,090 (72.9%)	396,559 (85.6%)	0.32	28,094 (93.5%)	28,186 (93.8%)	0.01
Number of hospital admissions^3^	3.28 (11.37)	2.16 (8.81)	0.11	4.16 (12.77)	4.81 (15.22)	0.05
Days since last hospital admission^3^	198 (110)	128 (125)	0.59	198 (111)	199 (112)	0.01
Any ED presentation	28,402 (68.8%)	281,592 (60.8%)	0.17	26,455 (88.0%)	24,920 (82.9%)	0.15
Number of ED presentations^3^	1.99 (3.19)	0.97 (1.47)	0.41	2.45 (2.65)	2.35 (3.82)	0.03
Days since last ED presentation^3^	191 (109)	125 (124)	0.57	192 (110)	188 (111)	0.04
**ED presentations by triage category (last 12 mo)** ^2^	*n* = 41,303	*n* = 463,173		*n* = 30,746	*n* = 30,746	
Non-urgent	3,369 (8.2%)	29,834 (6.4%)	0.07	3,150 (10.5%)	3,126 (10.4%)	0.00
Semi-urgent	13,074 (31.7%)	113,404 (24.5%)	0.16	12,140 (40.4%)	11,293 (37.6%)	0.06
Urgent	18,024 (43.6%)	133,136 (28.7%)	0.31	16,631 (55.3%)	15,288 (50.9%)	0.09
Emergency	12,835 (31.1%)	70,958 (15.3%)	0.38	11,976 (39.9%)	10,524 (35.0%)	0.10
Resuscitation	831 (2.0%)	4,843 (1.1%)	0.08	749 (2.5%)	780 (2.6%)	0.01
**Co-morbidities reported at last hospitalisation (last 12 mo)** ^2^	*n* = 30,090	*n* = 396,559		*n* = 28,094	*n* = 28,186	
Coronary artery disease	7,943 (26.4%)	52,643 (13.3%)	0.33	7,550 (26.9%)	6,925 (24.6%)	0.05
Congestive heart failure	3,275 (10.9%)	9,365 (2.4%)	0.35	3,012 (10.7%)	2,851 (10.1%)	0.02
Chronic obstructive pulmonary disease	4,915 (16.3%)	13,038 (3.3%)	0.45	4,266 (15.2%)	4,151 (14.7%)	0.01
Cardiovascular disease	20,201 (67.1%)	199,058 (50.2%)	0.35	18,982 (67.6%)	18,752 (66.5%)	0.02
Cerebrovascular disease	1,113 (3.7%)	18,015 (4.5%)	0.04	1,047 (3.7%)	1,348 (4.8%)	0.05
Dementia	615 (2.0%)	9,546 (2.4%)	0.03	565 (2.0%)	903 (3.2%)	0.08
Diabetes	5,946 (19.8%)	33,764 (8.5%)	0.33	5,277 (18.8%)	5,504 (19.5%)	0.02
Hypertension	657 (2.2%)	3,282 (0.8%)	0.11	535 (1.9%)	741 (2.6%)	0.05
Obesity	963 (3.2%)	7,377 (1.9%)	0.09	885 (3.2%)	927 (3.3%)	0.01
Renal disease	6,490 (21.6%)	46,109 (11.6%)	0.27	5,990 (21.3%)	6,156 (21.8%)	0.01
Respiratory disease	11,123 (37.0%)	62,674 (15.8%)	0.49	10,160 (36.2%)	9,706 (34.4%)	0.04
Smoking	5,769 (19.2%)	47,811 (12.1%)	0.20	5,289 (18.8%)	5,016 (17.8%)	0.03
**Number co-morbidities**						
0–2	9,872 (32.8%)	154,570 (39.0%)	0.13	9,238 (32.9%)	9,518 (33.8%)	0.02
3–5	11,244 (37.4%)	146,571 (37.0%)	0.01	10,512 (37.4%)	10,238 (36.3%)	0.02
6+	8,974 (29.8%)	95,418 (24.1%)	0.13	8,344 (29.7%)	8,430 (29.9%)	0.00

Data presented as number (percent) unless otherwise indicated.

^1^Age, sex, marital status, and Aboriginal status obtained at the most recent hospitalisation or ED presentation. For the CDMP evaluation cohort, this information was obtained from the CDMP minimum dataset (marital status not captured).

^2^For the CDMP evaluation cohort, the matched CDMP group, and the matched control group, service utilisation outcomes were obtained for the 3 or 12 mo preceding enrolment or matching. For potential controls, service utilisation outcomes were obtained by randomly selecting a 12-mo period between 1 January 2010 and 30 November 2013.

^3^Continuous variables summarised with mean (SD).

n/a, not available.

### Propensity Matching

Out of the 32,118 CDMP participants who had at least one hospital admission or ED presentation in the 12 mo preceding enrolment, 30,057 (73% of the CDMP evaluation cohort) were matched to a control participant. After matching, the majority of baseline characteristics became very well balanced between the two groups, with standardised differences smaller than 0.10, a level below which differences can be considered negligible [20] (Table 1). Exceptions include the proportion of patients with at least one unplanned hospitalisation in the previous 12 mo (SDiff = 0.13) and those with at least one ED presentation in the previous 12 mo (SDiff = 0.15).

**Fig 2:**
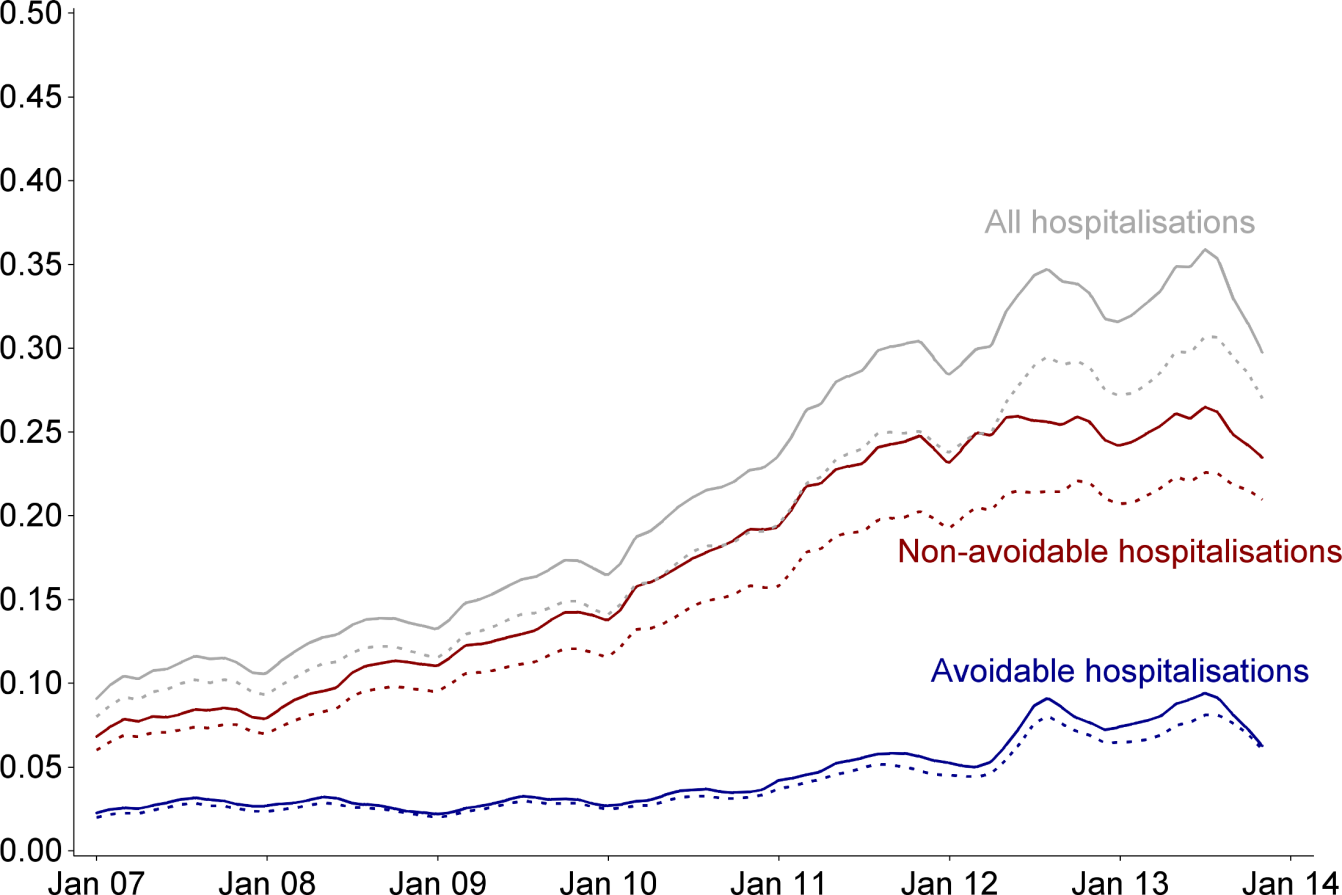
Average rate of hospitalisations per month in CDMP participants before and after matching. Monthly rate of hospitalisation per 100 patients. Dashed lines correspond to the entire CDMP evaluation cohort (*n* = 41,303); solid lines correspond to the matched CDMP group (*n* = 30,057). Denominators are participants who are alive. Curves were smoothed using a cubic spline with a 15% degree of smoothness.

Fig 2 shows the crude rate of avoidable, non-avoidable, and overall hospitalisations in the CDMP evaluation cohort, i.e., before matching (dashed lines), and in the matched CDMP group (solid lines). Patterns of avoidable hospitalisations before and after matching followed comparable trajectories over time, with both gradually increasing and experiencing peaks in the 2012 and 2013 winters. The rate of non-avoidable admissions before and after matching was also comparable; however, due to the greater number of events, there is a wider absolute gap between the two lines.

### Matched Analyses

Of the matched participants, 25,638 (85.3%) and 25,597 (85.2%) participants in the CDMP and control groups, respectively, were alive at the end of follow-up (chi-square *p* = 0.62). After adjustment for covariates, the adjusted relative risk of death was 0.96 (95% CI 0.91 to 1.01, *p* = 0.10). A post hoc analysis using an adjusted Cox model of time to death resulted in a hazard ratio of 0.99 (95% CI 0.95 to 1.03, *p* = 0.53).

**Fig 3:**
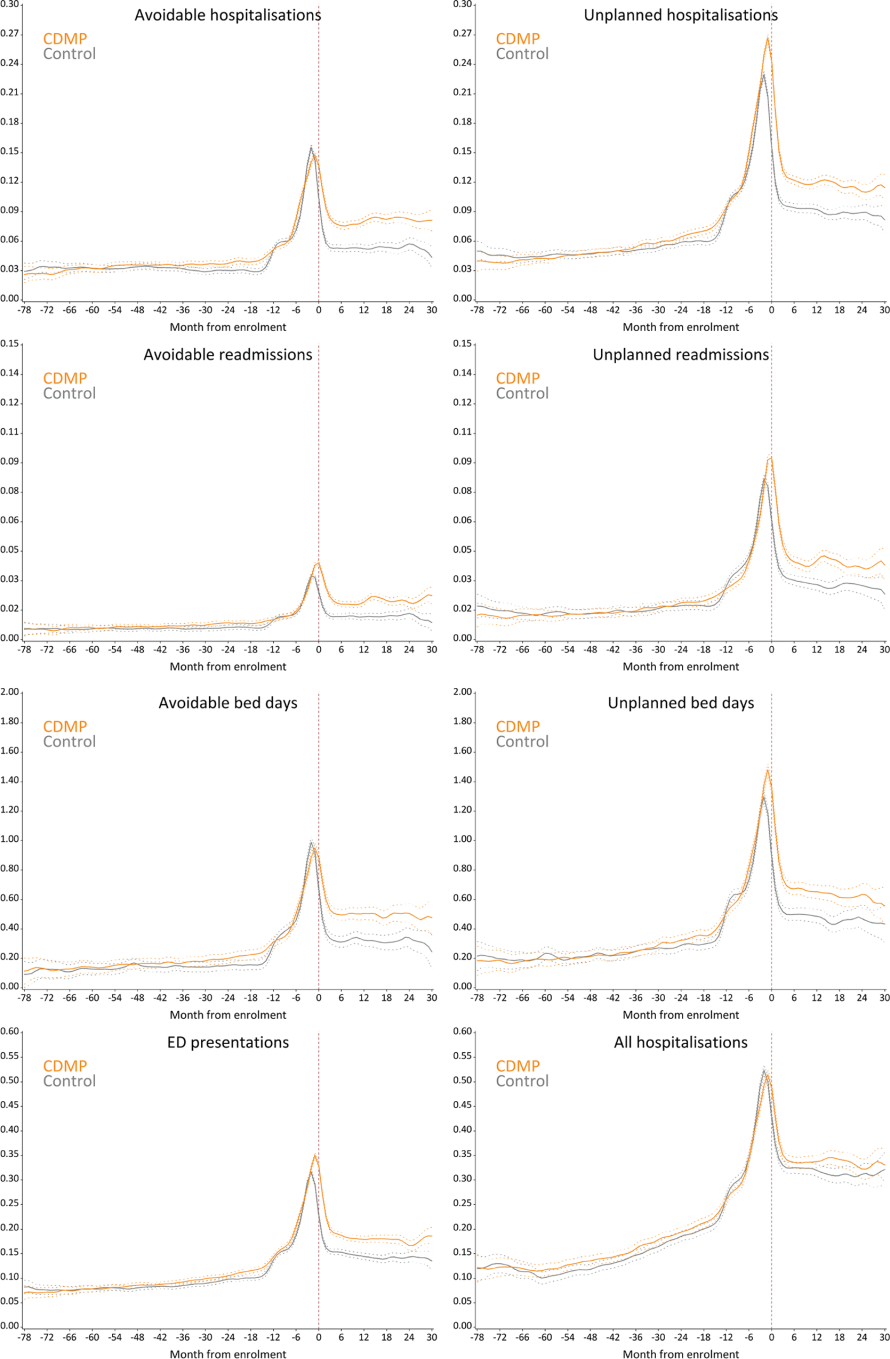
Monthly rate of health service utilisation after matching. Curves were smoothed using a cubic spline with a 15% degree of smoothness. Note varying vertical axis scales. *y*-Axes represent the average monthly event rate per patient.

Among survivors, the adjusted yearly rate of avoidable admissions between the month of enrolment and 31 December 2013 was 0.57 (95% CI 0.52 to 0.62) in the CDMP group versus 0.33 (95% CI 0.31 to 0.37) in the control group (Table 2), corresponding to an adjusted rate ratio of 1.70 (95% CI 1.62 to 1.79, *p <* 0.001). Depending on the month of enrolment/matching, the adjusted rate ratio varied between 1.14 and 2.65 (S1 Fig). Similar patterns were observed for other types of admissions and for length of stay measures. In particular, after enrolment, CDMP participants were at increased risk of ED presentations (rate ratio 1.37, 95% CI 1.32 to 1.42, *p <* 0.001) and had more bed days associated with avoidable admissions (rate ratio 1.70, 95% CI 1.59 to 1.82, *p <* 0.001). A post hoc analysis showed no evidence of a difference in the rate of non-avoidable hospitalisations (rate ratio 1.03, 95% CI 0.97 to 1.10, *p* = 0.26). Sensitivity analyses using a 2-y baseline as well as sensitivity analyses using multivariable models with no matching provided similar results (see S3 Table). Adjusted rates of health service utilisation over time (Fig 3) show a sharp increase in avoidable admissions in the months preceding enrolment in both the CDMP and control groups. Following enrolment, the rate of admission dropped in both groups; however, participants in the CDMP group remained at higher risk. A similar pattern was observed for avoidable bed days. Plots of unplanned admissions and ED presentations show a higher event rate in the CDMP group before enrolment/matching, which was maintained over the follow-up period.

**Table 2:** Result of matched analyses using Poisson regression.

Outcome	CDMP Group (*n* = 30,057/25,638^1^)	Control Group (*n* = 30,057/25,597^1^)	Risk or Rate Ratio	*p*-Value
**Unadjusted analysis**				
Death	14.7% (14.3%; 15.1%)	14.8% (14.4%; 15.3%)	0.99 (0.95; 1.03)	0.62
Avoidable hospitalisations	0.67 (0.65; 0.69)	0.41 (0.40; 0.43)	1.61 (1.53; 1.69)	<0.001
Non-avoidable hospitalisations	2.70 (2.51; 2.90)	3.24 (3.01; 3.48)	0.83 (0.75; 0.92)	<0.001
All hospitalisations	3.36 (3.17; 3.57)	3.65 (3.42; 3.90)	0.92 (0.84; 1.00)	0.062
Unplanned hospitalisations	1.12 (1.10; 1.14)	0.79 (0.76; 0.81)	1.42 (1.37; 1.48)	<0.001
ED presentations	1.77 (1.73; 1.82)	1.36 (1.31; 1.42)	1.30 (1.24; 1.36)	<0.001
Avoidable readmissions	0.14 (0.14; 0.15)	0.08 (0.07; 0.08)	1.90 (1.71; 2.11)	<0.001
Unplanned readmissions	0.33 (0.32; 0.35)	0.23 (0.22; 0.25)	1.44 (1.33; 1.56)	<0.001
Avoidable bed days	4.18 (4.02; 4.35)	2.65 (2.52; 2.79)	1.58 (1.48; 1.68)	<0.001
Unplanned bed days	5.94 (5.78; 6.10)	4.20 (4.06; 4.35)	1.41 (1.35; 1.48)	<0.001
**Adjusted analysis** ^**2**^				
Death	8.8% (8.1%; 9.5%)	9.2% (8.5%; 9.9%)	0.96 (0.91; 1.01)	0.10
Avoidable hospitalisations	0.57 (0.52; 0.62)	0.33 (0.31; 0.37)	1.70 (1.62; 1.79)	<0.001
Non-avoidable hospitalisations	1.44 (1.31; 1.59)	1.40 (1.27; 1.53)	1.03 (0.97; 1.10)	0.26
All hospitalisations	2.02 (1.87; 2.19)	1.80 (1.66; 1.95)	1.12 (1.07; 1.18)	<0.001
Unplanned hospitalisations	1.07 (1.01; 1.12)	0.75 (0.71; 0.79)	1.42 (1.37; 1.47)	<0.001
ED presentations	1.67 (1.57; 1.77)	1.22 (1.15; 1.29)	1.37 (1.32; 1.42)	<0.001
Avoidable readmissions	0.10 (0.09; 0.12)	0.05 (0.04; 0.06)	2.00 (1.80; 2.22)	<0.001
Unplanned readmissions	0.27 (0.24; 0.30)	0.18 (0.16; 0.20)	1.51 (1.40; 1.62)	<0.001
Avoidable bed days	3.35 (3.00; 3.75)	1.98 (1.76; 2.21)	1.70 (1.59; 1.82)	<0.001
Unplanned bed days	5.45 (5.05; 5.87)	3.82 (3.53; 4.13)	1.43 (1.36; 1.49)	<0.001

Data are presented as proportion (95% CI) and risk ratio (95% CI) for death, and yearly rate (95% CI) and rate ratio (95% CI) for service utilisation outcomes.

All analyses performed with Poisson regression with generalised estimating equations. For service utilisation outcomes, the number of years of follow-up is used as an offset to calculate yearly rates.

^1^The first sample size number indicates the number used for the analysis of death. The second sample size number indicates the number of survivors, as used for the analysis of all service utilisation outcomes.

^2^Adjusted for all covariates listed in S1 Text.

### Analysis of Hospitalisations by Primary Diagnosis

**Fig 4:**
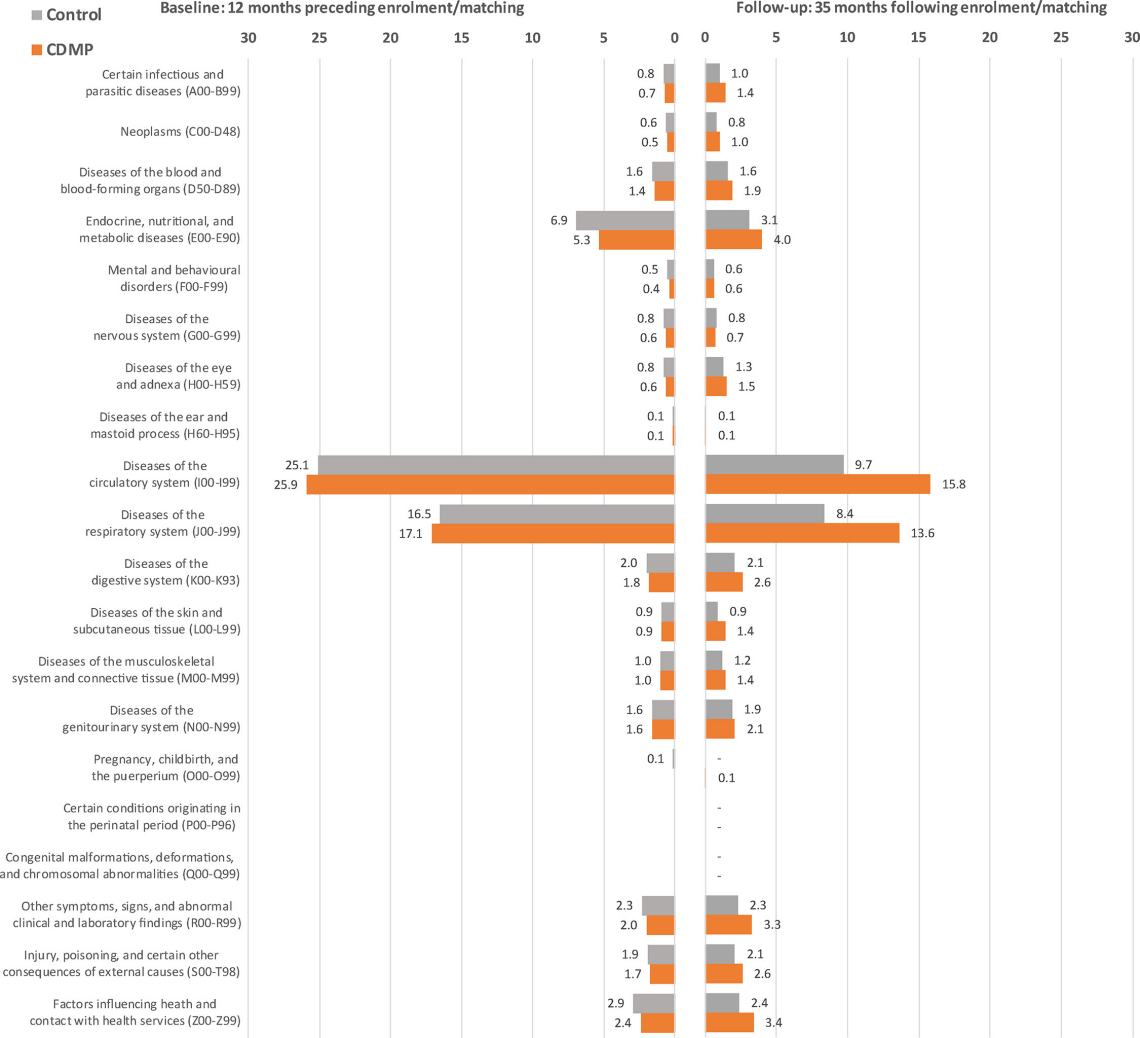
Breakdown of avoidable hospitalisations by ICD category before and after matching. Numbers indicate the percentage of participants experiencing at least one avoidable hospitalisation with a primary diagnosis in the corresponding ICD category.

A post hoc analysis breaking down hospitalisations by primary diagnosis was performed to investigate which diagnoses were linked to observed differences in avoidable hospitalisations. In the 12 mo preceding enrolment/matching, 51.2% and 51.4% of participants had at least one avoidable admission in the CDMP and control groups, respectively. During follow-up (median = 15 mo), 36.1% of CDMP participants and 26.2% of control participants had at least one avoidable admission. The breakdown by ICD category demonstrates good baseline balance between the two groups across all main diagnoses and that most of the excess seen in the CDMP group after enrolment/matching is driven by circulatory and respiratory diseases (see Fig 4).

## Discussion

Internationally, there is considerable interest in reducing the costs associated with the acute management of chronic diseases by focusing on strategies for better care in the community [4,5,22,23]. With older people and those with chronic disease utilising a significant proportion of health services in NSW, and accounting for almost half of total acute inpatient bed days, the CDMP represents a core feature of the large-scale and evolving system change by the NSW government to better connect the continuum of care. The CDMP was designed to better connect the care and support of people with chronic diseases who have been hospitalised or are at risk of avoidable and unplanned hospitalisation due to their chronic diseases. Specifically, the program aims to improve care and management in the community and lower avoidable hospitalisations. It builds on similar public health system innovations and their evaluation, for example in the UK [24], but is larger in scale, both in terms of the program itself and its evaluation.

We found no evidence of a general reduction in avoidable admissions, unplanned admissions, or ED presentations for patients enrolled in the CDMP compared to patients in the control group. In fact, compared to a matched control group, the CDMP group remained at higher risk of avoidable hospitalisation events following enrolment. However, there was no increase in the rate of non-avoidable admissions. This finding suggests that the excess in overall hospitalisation rates seen in the CDMP group is mainly driven by avoidable admissions, the majority of which appear to be related to conditions targeted by the CDMP, specifically respiratory and cardiovascular diagnoses. One possible explanation is that greater attention to patients enrolled in the program may at times have identified “unmet needs” judged to require admission to hospital. This has been similarly hypothesised elsewhere [24].

It is unclear whether the program had an effect on ED presentations and unplanned admissions due to residual imbalances after matching. Models suggest an excess that is consistent with the one observed for avoidable admissions; however, an analysis of adjusted rates over time shows that patterns of ED presentations and unplanned hospitalisations differed slightly and that the excess seen during follow-up may have been carried over from the pre-enrolment/matching period. A consistent feature of the findings was that, for all acute service outcomes, enrolment in the CDMP occurred at a peak in acute service utilisation. This implication presents a challenge for the program model, which tended to respond to realised risk (hospitalisation) rather than proactively identifying latent risks (e.g., risk factors and poor self-management) expected to impact future use of acute care. A similar effect has been reported by comparable programs, which have targeted public health systems [24]. In both the intervention and control groups, peaks in service utilisation were followed by a sharp reduction after enrolment. This outlines the natural tendency for high-risk patients to show reductions in use in the absence of any intervention, a phenomenon called “regression to the mean” reported in previous studies [24].

The results reported here need to be considered in the context of previous findings. Along with other recent similar international system-wide endeavours [24], we did not find evidence of reduced hospital use. Yet the evidence base supporting the interventions themselves is substantial, including several randomised trials [5,6,25]. An important feature of previous findings is that many of the positive findings tend to come from randomised studies while the negative findings seem to arise from the non-randomised studies. This potentially highlights the challenges associated with translating the results of well-controlled randomised trials to “business as usual” across an entire health system. Although the nature of the interventions remains fundamentally the same, the transition to the system-wide environment may dilute their impact through a variety of mechanisms: less tightly controlled recruitment criteria or variations in the way that self-management support and care coordination operate in different locations, for example. Where the interventions are more controlled and narrowly focused, system-wide interventions have been shown to demonstrate positive results using quasi-experimental methods such as those reported here [26].

A possible explanation for the contradictory nature of our findings may lie with fundamental differences in the implementation of the model of care for chronic disease management interventions between RCTs and system-wide interventions. The CDMP intervention was managed at two levels: the head office of NSW Health determined the core elements of the model of care (targeted enrolment, care coordination, and/or self-management support), and the local health districts determined the specific service model through which this was implemented. This sort of arrangement is common with system-wide interventions, particularly within decentralised systems. It has the merit of allowing the intervention to be made locally appropriate, but it can be more difficult to determine the precise services that clients were offered or received. Unfortunately, at the time of the evaluation, no data collection system was in place to record the details of the intervention, thus making it impossible to know exactly how the program was implemented for particular patients.

The major potential methodological limitation of this evaluation was the absence of a randomised control group. Having a randomised control group was not possible in this evaluation, where the intervention involved system-wide changes as well as services to individual patients. To overcome this limitation, a control group was constructed by matching CDMP participants to similar individuals in the NSW Admitted Patient Data Collection. A broad set of “potential controls” initially included anyone who had been hospitalised for any of the target conditions between 2007 and 2013 but had not been enrolled in the CDMP. Although the CDMP was open to everyone in NSW who was eligible, given the scale and complexity of the program, not everyone eligible had been enrolled in the CDMP by December 2013. This meant that potential controls included individuals who had not been enrolled but were otherwise eligible as well individuals who might have been offered the program but refused to participate. To ensure that controls used for the evaluation were similar to CDMP participants, we further restricted potential controls based on hospitalisation history and matched each CDMP participant to a control who had similar “baseline” characteristics at the time of CDMP enrolment using propensity scoring methods, thus aiming to replicate what would have happened in a randomised trial. Despite the matching and the covariate adjustments, we cannot completely exclude the presence of residual differences due to unmeasured confounders; however, given the absence of differences in all-cause mortality and non-avoidable hospitalisations, we believe systematic residual differences are unlikely to have occurred and to be so strong as to qualitatively affect the results.

We have also considered the potential limitations in our analyses due to selection bias. The statistical analyses necessarily concentrated on CDMP participants who were eligible for matching by having been admitted to hospital in the year preceding enrolment. This resulted in the analysis focusing on a majority of, but not all, CDMP participants. Post hoc analysis of the monthly rate of avoidable hospitalisations of matched and unmatched CDMP participants indicated that the overall pattern was similar. This analysis confirms that the selection of individuals through the matching process is not likely to have systematically changed the outcomes of the analyses or the conclusions of the evaluation. The results of this evaluation reflect a multifaceted intervention implemented by the public health services of NSW. Although each health system is unique, many of the challenges associated with the implementation and evaluation of the CDMP are likely to be applicable to other health systems in industrialised countries faced with increasing numbers of individuals with chronic conditions, as has been shown by similar programs in the UK [27].

A possible issue is the choice of an appropriate outcome measure for programs like the CDMP. By focusing on people with a history of hospital admissions, the programs here and elsewhere have concentrated on patients more likely to demonstrably benefit from the intervention in the short term; however, as a sole outcome suite, acute service use may not be able to fully capture the effect of these programs. Additional data—for example, quality of life, number of general practice visits, or the intensity of social care use—could provide more sensitive measures.

In conclusion, participation in the CDMP was associated with an increase in avoidable or “potentially preventable” health service utilisation compared to matched controls, but there was no difference in mortality or in non-avoidable hospitalisations. A possible explanation is the presence of unmet needs identified by the program. Service utilisation decreased sharply following its peak for both groups. This finding reflects the tendency for high-risk patients to show reductions in service use in the absence of any specific intervention and underscores the need for an appropriate control group when assessing the effect of interventions on hospital use. It also suggests that future programs should intervene with patients at a time when they are most able to benefit from extra care, rather than when their service use has reached a peak. This may require the focus of recruitment and intervention to shift from hospital services to primary care, where high levels of need may first be detected and early intervention is possible.

## Supporting Information

S1 Fig Effect of the intervention on avoidable hospitalisations by month of enrolment/matching. (TIF)

S1 File Prespecified statistical analysis plan. (PDF)

S1 Table Details of the intervention. (DOCX)

S2 Table Definition of avoidable hospitalisations. (DOCX)

S3 Table Sensitivity analyses using a 2-y baseline and using a randomly selected set of control records. (DOCX)

S1 Text List of covariates. (DOCX)

## Abbreviations

CDMP: Chronic Disease Management Program
ED: emergency department
NSW: New South Wales
NSW: Health, New South Wales Health
RCT: randomised controlled trial
SD: standard deviation
SDiff: standardised difference
