# Supplementary figures and images for: Impact Evaluation of a System-Wide Chronic Disease Management Program on Health Service Utilisation: A Propensity-Matched Cohort Study
Source: PLoS Med. 2016 Jun 7;13(6):e1002035. doi: 10.1371/journal.pmed.1002035 (PMC4896436; doi:10.1371/journal.pmed.1002035)

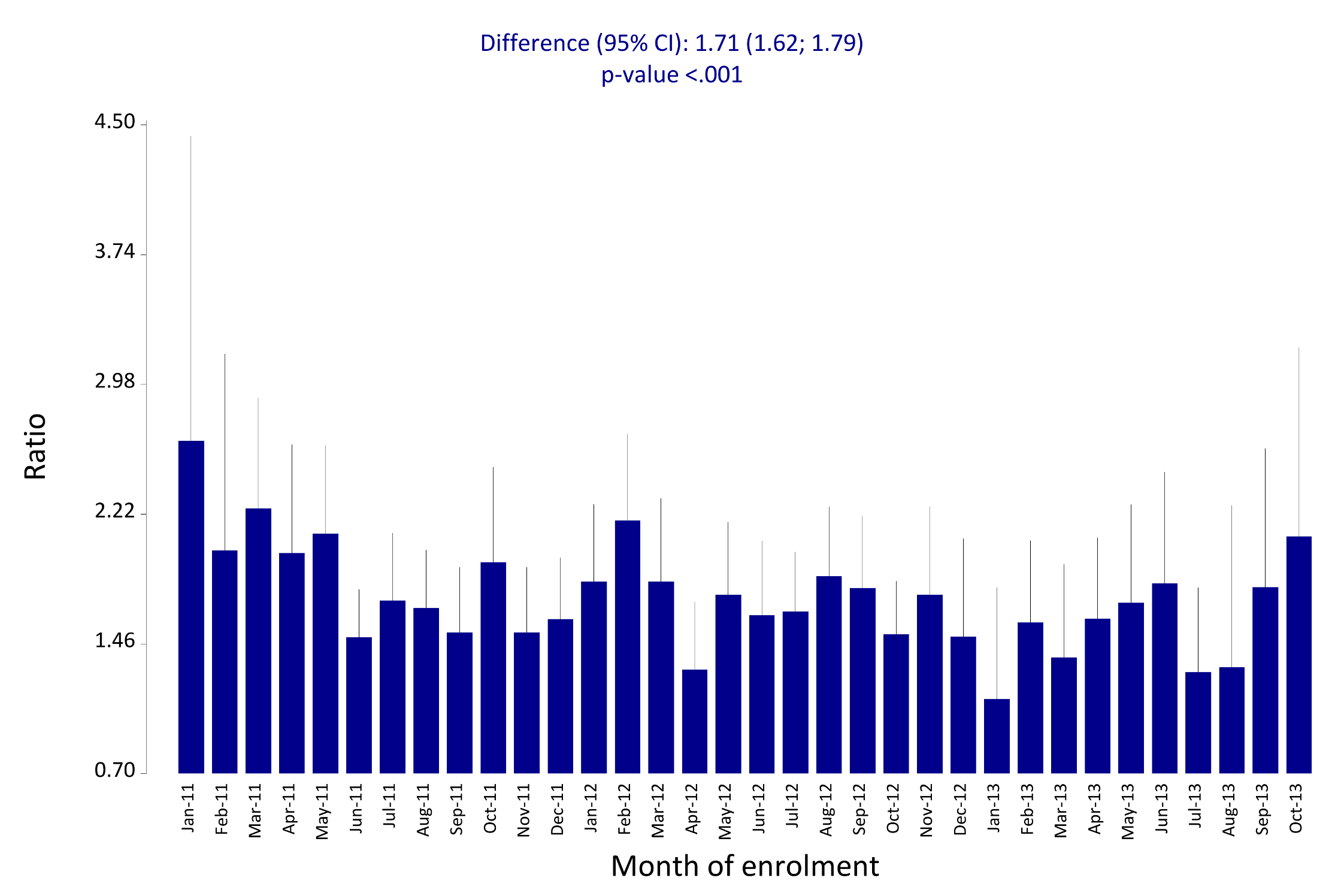

Supplement: S1 Fig — (TIF) [file pmed.1002035.s001.tif]
