# Supplementary material for: Impact Evaluation of a System-Wide Chronic Disease Management Program on Health Service Utilisation: A Propensity-Matched Cohort Study
Source: PLoS Med. 2016 Jun 7;13(6):e1002035. doi: 10.1371/journal.pmed.1002035 (PMC4896436; doi:10.1371/journal.pmed.1002035)
Supplement: S1 File — (PDF) [file pmed.1002035.s002.pdf]

Statistical Analysis Plan

CDMP quantitative evaluation

1 Data sources

1.1 The Chronic Disease Management Program Minimum Data Set

The analysis will include every participant recorded in the program minimum dataset and enrolled between January 2011 and December 2013. The date of enrolment will be calculated using the following approach: If the Program consent date is available, it will be used as the enrolment date. If it is missing, and the Enrolment decision date is available, then this date will be used. If this is also not available, then the Registration date will be used as enrolment date instead.

1.2 APDC

The APDC dataset will contain every public and private hospital admission which occurring between January 2007 and December 2013 in NSW. CDMP participants will be flagged.

1.3 EDDC

The APDC dataset will contain every public and private emergency department admission occurring between January 2007 and December 2013 in NSW. CDMP participants will be flagged.

1.4 Registry of birth, deaths and marriages (RBDM)

The RBDM dataset will contain every death recorded in NSW between January 2007 and December 2013 in NSW. CDMP participants will be flagged.

2 Subject disposition and baseline characteristics

2.1 Subject Disposition

A bar chart will show the number of patient registrations in the CDMP outcomes register by 6-month period for the entire duration of the program i.e. between January 2009 and December 2013.

2.2 Description of CDMP participants

We will describe baseline characteristics of CDMP participants overall and broken down by year of enrolment (2011, 2012 and 2013). Baseline characteristics will include:

- Age
- Gender
- Aboriginality
- ARIA
- SEIFA

- Source of referral
- Target condition(s)
- Risk of hospitalisation
- Special population flag
- Initial SF1
- Initial intervention
- Reasons for non-enrolment
- Referral target
- Program consent
- Data use consent
- Number of hospitalisations in 2010, average length of hospital stay, total hospital bed days
- Number of unplanned hospitalisations in 2010, average length of unplanned hospital stay, total unplanned hospital bed days
- Number of avoidable hospitalisations in 2010, average length of avoidable hospital stay, total avoidable hospital bed days
- Number of ED admissions in 2010 overall and by triage categories 1-5

3 Evaluation of efficacy outcomes

3.1 Efficacy outcomes

The primary efficacy outcome will be the rate of avoidable hospitalisations. Avoidable hospitalisations are defined as hospitalisations that fall under any of the following categories:

1. Vaccine-preventable:
    - "Influenza and pneumonia" (where any of the diagnosis fields have ICD-10-AM code as J10, J11, J13, J14, J15.3, J15.4, J15.7, J15.9, J16.8, J18.1, J18.8 excluding those where secondary diagnosis is D57)
    - "Other preventable vaccine" (where any of the diagnosis fields have ICD-10-AM code as A35, A36, A37, A80, B05, B06, B16.1, B16.9, B18.0, B18.1, B26, G00.0, M01.4)
2. Chronic:
    - "Diabetes complications" (where any of the diagnosis fields have ICD-10-AM code as E10.1-E10.8, E11.0-E11.8, E13.0-E13.8, E14.0-E14.8)
    - "Nutritional deficiencies" (where principal diagnosis has ICD-10-AM code as any of E40-E43, E55.0, E64.3)
    - "Iron deficiency anaemia" (where principal diagnosis has ICD-10-AM code as any of D50.1-D50.9)
    - "Hypertension" (where principal diagnosis has ICD-10-AM code as any of I10, I11.9)
    - "Congestive heart failure" (where principal diagnosis has ICD-10-AM code as any of I11.0, I50, J81)
    - "Angina" (where principal diagnosis has ICD-10-AM code as any of I20, I24.0, I24.8, I24.9)
    - "Chronic obstructive pulmonary disease" (where principal diagnosis has ICD-10-AM code as any of J41-J44, J47 or the principal diagnosis ICD-10-AM code is J20 with secondary diagnosis as J41, J42, J43, J44, J47)

- "Asthma" (where principal diagnosis has ICD-10-AM code as any of J45, J46)

3. Acute:
   - "Dehydration and gastroenteritis" (where principal diagnosis has ICD-10-AM code as any of E86, K52.2, K52.8, K52.9)
   - "Convulsions and epilepsy" (where principal diagnosis has ICD-10-AM code as any of G40, G41, O15, R56)
   - "Ear, nose and throat infections" (where principal diagnosis has ICD-10-AM code as any of H66, H67, J02, J03, J06, J31.2)
   - "Dental conditions" (where principal diagnosis has ICD-10-AM code as any of A69.0, K02-K06, K08, K09.8, K09.9, K12, K13)
   - "Perforated/bleeding ulcer" (where principal diagnosis has ICD-10-AM code as any of K25.0-K25.2, K25.4-K25.6, K26.0-K26.2, K26.4-K26.6, K27.0-K27.2, K27.4-K27.6, K28.0-K28.2, K28.4-K28.6)
   - "Ruptured appendix" (where any of the diagnosis fields have ICD-10-AM code as K35.0)
   - "Pyelonephritis" (where principal diagnosis has ICD-10-AM code as any of N10, N11, N12, N13.6)
   - "Pelvic inflammatory disease" (where principal diagnosis has ICD-10-AM code as any of N70, N73, N74)
   - "Cellulitis" (where principal diagnosis has ICD-10-AM code as any of L03, L04, L08.0, L08.8, L08.9, L88, L98.0, L98.3)
   - "Gangrene" (where any of the diagnosis fields have ICD-10-AM code as R02)

Secondary efficacy outcomes will include:

1. Unplanned hospitalisations defined as hospitalisations that have status of “emergency” or the mode of separation is not “Transfer to Palliative Care Unit / Hospice” or any of the secondary diagnoses are not “Palliative care “(Z51.5)”.
2. Hospital re-admissions defined as admissions occurring less than 30 days after discharge
3. Emergency department admissions
4. Deaths
5. Number of hospital bed-days

3.2 Analysis of the CDMP cohort

3.2.1 Utilisation patterns over-time

For outcomes defined as number of events (hospital and ED admissions), we will calculate rates and their 95% CI for every 6-month period between January 2011 and December 2013. Death will be considered as a censoring event i.e. if a participant dies after four months in a given semester, the event rate will only be calculated over 4 months.

Results will be presented on a plot, first for all program participants together and then separately depending on the enrolment semester to identify potentially different patterns between those enrolled early vs late into the program.

3.2.2 *Before-after analyses*

Before-after analyses using will compare the rate of hospitalisations before enrolment into the program to the rate after enrolment into the program. For the before-after comparison, only patients enrolled between January 11 and June 13 will be included while only counting events occurring between 1 July 2010 (6 months before January 11) and 31 December 2013 (6 months after June 13).

For the purpose of this analysis every program participant will therefore contribute 2 data points; one before enrolment and one after enrolment. The analysis will be a Poisson regression with the period (before vs after) as a fixed effect. Generalized estimating equations with a compound symmetry structure will be used to model the correlations between multiple measurements from each patient.

3.2.3 *Time-dependent analyses*

Time-dependent analyses will be used to assess the impact of “exposure to the program” on the rate of hospitalisations. Exposure to the program will be defined for each semester as the proportion of the time a participant was exposed to the program e.g. if a patient gets enrolled into the program halfway through a semester, his/her exposure will be 50% (s/he was on the program 50% of the time during that particular semester).

The time-dependent analysis will cover a total of 6 semesters. All patients enrolled between January 2011 and December 2013 will be included and all events occurring within the same timeframe will be considered. For the purpose of this analysis every program participant will therefore contribute 6 data points; one per semester. The analysis will be a Poisson regression with the exposure proportion as a fixed effect. A sensitivity analysis will also include the semester and its interaction with exposure. Generalized estimating equations with a compound symmetry structure will be used to model the correlations between multiple measurements from each patient

3.2.4 *Adjusted analyses*

Both the before-after and the time-dependent analyses will be performed without and with adjustment. Potential covariates will include LHD, age, gender, aboriginality, ARIA, SEIFA, target conditions, SF1 and previous patterns of service utilisation (number of hospitalisations in 2010, number of unplanned hospitalisations in 2010, number of avoidable hospitalisations in 2010 and number of ED admissions in 2010).

Each potential covariate will first be analysed using a univariate Poisson regression and only those with a univariate p-value smaller than 0.20 will be included in the final model. To avoid problems due to colinearity, in case of two covariates with a Pearson correlation coefficient greater than 0.8, only the one with the smallest univariate p-value will be kept in the final model. For covariates with more than 10% of data missing, we will create a separate category labeled “missing”.

3.3 Analyses using a control group

3.3.1 *Matched analyses*

The primary analysis for assessing the effect of the program will be performed using a propensity-matched cohort as the comparator.

Propensity scoring and matching

Using baseline characteristics, we will calculate a propensity score defined as the probability of getting enrolled in the program. Matched controls will be selected from the APDC dataset using individuals who never got enrolled into the program. We will follow the following steps:

Step 1: Group program participants by period (e.g. month/quarter/semester) according to their enrolment date (the final "grouping period" will depend on the number of participants recruited).

Step 2: For each stratum/period, obtain details of the latest hospitalisation occurring in the 12 months preceding the period. Any program participant with no hospitalisation in the preceding 12 months will be excluded from propensity-based analyses.

Step 3: Within each stratum/period, calculate propensity score using a logistic regression modeling the probability of getting enrolled in the program. Covariates will include all variables considered for adjusted analyses.

Step 4: Within each stratum/period, starting with the earliest period, match 2 non-CDMP participants for each CDMP participant on the propensity score using optimal matching procedures. A control which has been used as a match in an earlier period is no longer available as match for a future period.

Step 6: Check the quality of the matching by plotting the propensity score distribution between the cases and controls overall and by period and by calculating the standardised difference for every covariate included in the propensity score calculation.

Analysis of matched data

The matched set will be analysed using Poisson regression with generalized estimating equations to account for the matched-nature of the data. The outcome will be the event rate (e.g. rate of avoidable hospitalisations) occurring after the "matching-period".

Sensitivity analyses

Sensitivity analyses using different baseline periods (e.g. 6 months or 2 years) will be performed to assess the impact on the size of the matched cohort as well as the results.
