# Supplementary material for: Impact Evaluation of a System-Wide Chronic Disease Management Program on Health Service Utilisation: A Propensity-Matched Cohort Study
Source: PLoS Med. 2016 Jun 7;13(6):e1002035. doi: 10.1371/journal.pmed.1002035 (PMC4896436; doi:10.1371/journal.pmed.1002035)
Supplement: S1 Table — (DOCX) [file pmed.1002035.s003.docx]

| **Program elements** | **CDMP Service Model** |
| --- | --- |
| **Targeted enrolment** | - The target population for the CDMP are people aged 16 years or over who are at very high, or high risk, of hospitalisation and have been diagnosed with at least one of five chronic conditions: diabetes, congestive heart failure, coronary artery disease, chronic obstructive disease, and hypertension. - Individuals are enrolled by a trained health care professional, usually using a risk assessment algorithm. - Priority populations for the Program are Aboriginal people and frail elderly people, as well as culturally and linguistically diverse communities and people with serious mental illness where individuals are at high or very high risk of hospitalisation. - Participants provide consent to be enrolled into the Program, and to have their information used in the evaluation. |
| **Comprehensive assessment** | - A Program staff member or other health care professional, for example the participants general practitioner, is responsible for the development of a comprehensive assessment which will address all of the facets of a person’s life relevant to the decision-making such as the development of care plans and/or referrals to services. |
| **Shared care planning** | - All Program participants enrolled in the CDMP will have a shared care plan – developed in consultation with the participant - that is based on the comprehensive assessment. - The shared care plan will vary from person to person depending on the individual needs. |
| **Continuum of care coordination** | - Effective care coordination is a critical element of chronic disease management and therefore the CDMP. - Care coordination involves the delivery of services by different providers occurs in a coherent, logical and timely manner, consistent with the person’s medical needs and personal context. |
| **Self-management support** | - The Program will also provide self-management support to participants through health coaching and other support services. - The self-management support services provided to participants will depend on local infrastructure and capacity and may involve support through individual or group education with an active goal setting component, health coaching or motivational interviewing by telephone or in person, monitoring symptoms with technology, coaching with proactive goal setting and follow up, provision of information, and programs based on psychological and emotional support that acknowledges people’s stages of change. |
| **Scheduled monitoring and review** | - A data review will be specified in the Program participants shared care plan. - Local systems will be in place to ensure review occurs and necessary follow up action is initiated. |
