# Supplementary material for: Impact Evaluation of a System-Wide Chronic Disease Management Program on Health Service Utilisation: A Propensity-Matched Cohort Study
Source: PLoS Med. 2016 Jun 7;13(6):e1002035. doi: 10.1371/journal.pmed.1002035 (PMC4896436; doi:10.1371/journal.pmed.1002035)
Supplement: S2 Table — (DOCX) [file pmed.1002035.s004.docx]

| **Category** | **ICD-10-AM (2nd edition) codes** | **Additional selection information** |
| --- | --- | --- |
| **Vaccine-preventable** |  |  |
| Influenza and pneumonia | J10, J11, J13, J14, J15.3, J15.4, J15.7, J15.9, J16.8, J18.1, J18.8 | In any diagnosis field; exclude people under 2 months; ICD-10-AM: exclude cases with secondary diagnosis of D57 |
| Other vaccine preventable | A35, A36, A37, A80, B05, B06, B16.1, B16.9, B18.0, B18.1, B26, G00.0, M01.4 | In any diagnosis field |
| **Chronic** |  |  |
| Diabetes complications | E10.1-E10.8, E11.0-E11.8, E13.0- E13.8, E14.0-E14.8 | In any diagnosis field |
| Nutritional deficiencies | E40-E43, E55.0, E64.3 | Principal diagnosis only |
| Iron deficiency anaemia | D50.1-D50.9 | Principal diagnosis only |
| Hypertension | I10, I11.9 | Principal diagnosis only; ICD-10-AM: exclude cases with procedures in blocks 600-693, 705-707, 717 and procedure codes 38721-00, 38721-01, 90226-00 |
| Congestive heart failure | I11.0, I50, J81 | Principal diagnosis only; ICD-10-AM: exclude cases with procedures in blocks 600-693, 705-707, 717 and procedure codes 38721-00, 38721-01, 90226-00 |
| Angina | I20, I24.0, I24.8, I24.9 | Principal diagnosis only; ICD-10-AM: exclude cases with procedure codes in blocks 1-1779 |
| Chronic obstructive pulmonary disease | J41-J44, J47, (J20) | Principal diagnosis only; ICD-10-AM: J20 only with second diagnosis of J41, J42, J43, J44, J47 |
| Asthma | J45, J46 | Principal diagnosis only |
| **Acute** |  |  |
| Dehydration and gastroenteritis | E86, K52.2, K52.8, K52.9 | Principal diagnosis only |
| Convulsions and epilepsy | G40, G41, O15, R56 | Principal diagnosis only |
| Ear, nose and throat infections | H66, H67, J02, J03, J06, J31.2 | Principal diagnosis only |
| Dental conditions | A69.0, K02-K06, K08, K09.8, K09.9, K12, K13 | Principal diagnosis only |
| Perforated/bleeding ulcer | K25.0- K25.2, K25.4-K25.6, K26.0- K26.2, K26.4-K26.6, K27.0-K27.2, K27.4-K27.6, K28.0-K28.2, K28.4- K28.6 | Principal diagnosis only |
| Ruptured appendix | K35.0 | In any diagnosis field |
| Pyelonephritis | N10, N11, N12, N13.6 | Principal diagnosis only |
| Pelvic inflammatory disease | N70, N73, N74 | Principal diagnosis only |
| Cellulitis | L03, L04, L08.0, L08.8, L08.9, L88, L98.0, L98.3 | ICD-10-AM: Include cases where L03, L04, L08.0, L08.8, L08.9, L88, L98.0, L98.3 is reported as the principal diagnosis AND there were either no procedures reported OR the procedures listed were only in blocks 1604- 1606, 1608, 1820-2016 or the procedures 90660-00, 30207-00, 30676-00, 30679-00, 34530-01 and 47912-00 Additionally, check that the procedure is the only procedure when in the list: blocks 1604-1606, 1608, or the procedures are: 90660-00, 30207-00, 30676-00, 30679-00, 34530-01 and 47912-00] |
| Gangrene | R02 | In any diagnosis field |

Note: This list is based on the Australian work by Vic DHS and subsequent development by NSW Health: the method of simplifying the procedure exclusions in ICD-10 by the use of procedure code blocks was developed by NSW Health.
