# Supplementary material for: Impact Evaluation of a System-Wide Chronic Disease Management Program on Health Service Utilisation: A Propensity-Matched Cohort Study
Source: PLoS Med. 2016 Jun 7;13(6):e1002035. doi: 10.1371/journal.pmed.1002035 (PMC4896436; doi:10.1371/journal.pmed.1002035)
Supplement: S3 Table — (DOCX) [file pmed.1002035.s005.docx]

**S3. Sensitivity analyses using a 2-year baseline and a multivariable model**

|  | **Proportions or yearly rates ^1^** | | **Risk or rate** |  |
| --- | --- | --- | --- | --- |
| **Outcome** | **CDMP** | **Control** | **ratio (95% CI)** | **P-value** |
|  |  |  |  |  |
| **Multivariable analysis without matching using a 1-year baseline** | | | | |
|  | **N=32,070/26,517 ^2^** | **N=460,377/364,765 ^2^** |  |  |
| Death | 8.8% (8.4%; 9.2%) | 11.7% (11.3%; 12.1%) | 0.75 (0.73; 0.78) | <.001 |
| Avoidable hospitalisations | 0.42 (0.41; 0.43) | 0.19 (0.18; 0.19) | 2.24 (2.21; 2.28) | <.001 |
| Non-avoidable hospitalisations | 1.24 (1.23; 1.25) | 1.01 (1.00; 1.01) | 1.23 (1.22; 1.24) | <.001 |
| All hospitalisations | 1.64 (1.63; 1.66) | 1.22 (1.21; 1.22) | 1.35 (1.34; 1.36) | <.001 |
| Unplanned hospitalisations | 0.92 (0.90; 0.93) | 0.49 (0.48; 0.49) | 1.88 (1.86; 1.91) | <.001 |
| ED admissions | 1.36 (1.34; 1.37) | 0.76 (0.75; 0.76) | 1.80 (1.78; 1.81) | <.001 |
| Avoidable readmissions | 0.068 (0.065; 0.073) | 0.023 (0.021; 0.024) | 3.03 (2.91; 3.15) | <.001 |
| Unplanned readmissions | 0.22 (0.22; 0.23) | 0.10 (0.10; 0.11) | 2.18 (2.13; 2.23) | <.001 |
| Avoidable bed days | 2.36 (2.34; 2.38) | 1.04 (1.03; 1.05) | 2.27 (2.26; 2.29) | <.001 |
| Unplanned bed days | 4.52 (4.48; 4.55) | 2.40 (2.38; 2.41) | 1.88 (1.87; 1.89) | <.001 |
|  |  |  |  |  |
| **Matched analysis using a 2-year baseline** | | | | |
|  | **N=33,761/28,937 ^2^** | **N=33,761/29,222 ^2^** |  |  |
| Death | 8.5% (7.8%; 9.2%) | 8.4% (7.7%; 9.1%) | 1.01 (0.96; 1.06) | 0.80 |
| Avoidable hospitalisations | 0.45 (0.41; 0.48) | 0.27 (0.25; 0.29) | 1.66 (1.58; 1.75) | <.001 |
| Non-avoidable hospitalisations | 1.35 (1.20; 1.51) | 1.25 (1.12; 1.41) | 1.07 (1.01; 1.14) | 0.020 |
| All hospitalisations | 1.81 (1.65; 2.00) | 1.57 (1.42; 1.73) | 1.16 (1.10; 1;22) | <.001 |
| Unplanned hospitalisations | 0.88 (0.84; 0.93) | 0.62 (0.59; 0.65) | 1.43 (1.39; 1.48) | <.001 |
| ED admissions | 1.36 (1.29; 1.44) | 1.03 (0.97; 1.09) | 1.32 (1.28; 1.37) | <.001 |
| Avoidable readmissions | 0.066 (0.056; 0.077) | 0.034 (0.029; 0.040) | 1.94 (1.76; 2.13) | <.001 |
| Unplanned readmissions | 0.20 (0.17; 0.22) | 0.13 (0.11; 0.15) | 1.52 (1.41; 1.63) | <.001 |
| Avoidable bed days | 2.67 (2.36; 3.02) | 1.56 (1.38; 1.76) | 1.71 (1.61; 1.82) | <.001 |
| Unplanned bed days | 4.57 (4.25; 4.91) | 3.08 (2.85; 3.32) | 1.49 (1.42; 1.55) | <.001 |
|  |  |  |  |  |

**Notes:**

1. Proportions and relative risk for death. Yearly rate and rate ratio for service utilisation outcomes

2. The first number indicates the number used for the analysis of death. The second number indicates the number of survivors used for the analysis of all service utilisation outcomes.

All analyses performed with Poisson regression with generalised estimating equations. For service utlisation outcomes, years of follow-up is used as an offset to calculate yearly rates.

Adjusted for all covariates listed in Text S1
