# Supplementary material for: Impact Evaluation of a System-Wide Chronic Disease Management Program on Health Service Utilisation: A Propensity-Matched Cohort Study
Source: PLoS Med. 2016 Jun 7;13(6):e1002035. doi: 10.1371/journal.pmed.1002035 (PMC4896436; doi:10.1371/journal.pmed.1002035)
Supplement: S1 Text — (DOCX) [file pmed.1002035.s006.docx]

**List of covariates included in propensity score calculation and in adjusted analyses**

- **Socio-demographic characteristics**

1. Age (years)
2. Sex (Male, Female)
3. Indigenous status (Yes, No)
4. Marital status (Divorced/permanently separated, Married/de facto, Never married/single, Windowed, Unknown/missing)
5. Area health service (Greater Southern, Greater Western, Hunter New England, North Coast, Northern Sydney / Central Coast, South Eastern Sydney / Illawarra, Sydney South West, Sydney West, St Vincent's Health Network)

- **Service utilisation history**

1. Number of hospitalisations in the last 12 months
2. Number of hospitalisations in the last 3 months
3. Number of unplanned hospitalisations in the last 12 months
4. Number of avoidable hospitalisations in the last 12 months
5. Number of avoidable hospitalisations in the last 3 months
6. Number of avoidable bed days in the last 12 months
7. Number of bed days in the last 12 months
8. Number of unplanned bed days in the last 12 months
9. Number of ED presentations in the last 12 months
10. Number of ED presentations in the last 3 months
11. Flag for ED presentation in the resuscitation category in the last 12 months (yes/no)
12. Flag for ED presentation in the emergency category in the last 12 months (yes/no)
13. Flag for ED presentation in the urgent category in the last 12 months (yes/no)
14. Flag for ED presentation in the semi-urgent category in the last 12 months (yes/no)
15. Flag for ED presentation in the non-urgent category in the last 12 months (yes/no)
16. Number of days since the last ED presentation (0 - 30 days, <30 - 90 days, <90 - 180 days, <180 - 360 days, <360 days)
17. Number of days since the last hospital admission (0 - 30 days, <30 - 90 days, <90 - 180 days, <180 - 360 days, <360 days)

- **Flags (yes/no) recording the occurrence of hospitalisations in the last 12 months with primary diagnosis in of the following ICD categories:**

1. Certain infectious and parasitic diseases (A00-B99)
2. Neoplasms (C00-D48)
3. Diseases of the blood and blood-forming organs (D50-D89)
4. Endocrine, nutritional, and metabolic diseases (E00-E90)
5. Mental and behavioural disorders (F00-F99)
6. Diseases of the nervous system (G00-G99)
7. Diseases of the eye and adnexa (H00-H59)
8. Diseases of the ear and mastoid process (H60-H95)
9. Diseases of the circulatory system (I00-I99)
10. Diseases of the respiratory system (J00-J99)
11. Diseases of the digestive system (K00-K93)
12. Diseases of the skin and subcutaneous tissue (L00-L99)
13. Diseases of the muscoloskeletal system and connective tissue (M00-M99)
14. Diseases of the genitourinary system (N00-N99)
15. Pregnancy, childbirth, and the puerperium (O00-O99)
16. Congenital malformations, deformations, and chromosomal abnormalities (Q00-Q99)
17. Other symptoms, signs, and abnormal clinical and laboratory findings (R00-R99)
18. Injury, poisoning, and certain other consequences of external causes (S00-T98)
19. Factors influencing heath and contact with health services (Z00-Z99)

- **Flags for conditions targeted by the program as recorded during hospitalisations over the last 12 months**

1. COPD as primary diagnosis (yes/no)
2. Coronary artery disease as primary diagnosis (yes/no)
3. Hypertension as primary diagnosis (yes/no)
4. Congestive heart failure as primary diagnosis (yes/no)
5. Diagnosis of diabetes (yes/no)

- **Other important co-morbidities recorded during hospitalisations over the last 12 months**

1. Cardiovascular disease (yes/no)
2. Cerebrovascular disease (yes/no)
3. Dementia (yes/no)
4. Obesity (yes/no)
5. Renal disease (yes/no)
6. Respiratory disease (yes/no)
7. Smoking (yes/no)
8. Total number of co-morbidities (not hospitalised in last 12 months, 0-2, 3-5, 6+)

- **Extra variables included in the adjusted analyses but not in the propensity score calculation**

1. Propensity score
2. Month of enrolment (1 to 34)
3. Flag for any avoidable hospitalisation in the last 12 months (yes/no)
4. Flag for any unplanned hospitalisation in the last 12 months (yes/no)
5. Flag for any ED presentation in the last 12 months (yes/no)
